# Special Issue—“Natural Products That Might Change Society”

**DOI:** 10.3390/molecules29051008

**Published:** 2024-02-26

**Authors:** Yhiya Amen, Kuniyoshi Shimizu

**Affiliations:** 1 Department of Pharmacognosy, Faculty of Pharmacy, Mansoura University, Mansoura 35516, Egypt; yhiaamen@mans.edu.eg; 2 Department of Agro-Environmental Sciences, Graduate School of Bioresources and Bioenvironmental Sciences, Kyushu University, Fukuoka 819-0395, Japan

This Special Issue of *Molecules* gathers eight research papers and two review articles covering the isolation, identification, and biological activity of selected natural products, with the aim of discovering potential candidates that could change society and improve human health. Natural products and their analogues play a very important role in the drug-discovery process [1]. They represent a major source of new chemical entities with potential biological activities. The discovery of new bioactive natural products as leads can indeed change society and provide a significant positive impact on humanity. Numerous natural products throughout history have had a profound impact on society, and really shaped human history and influenced various aspects of our lives [2]. In this Special Issue, the plants covered are *Artemisia judaica, Jasminum humile*, *Jasminum grandiflorum, Pelargonium × hortorum*, *Pelargonium sidoides*, *Phyllostachys pubescens*, *Retama monosperma*, and *Xanthium spinosum*. In addition, some articles cover endophytic fungi, seaweeds, and mushrooms.

The first published article (Contribution 1) in this Special Issue focuses on *Jasminum humile* and *Jasminum grandiflorum* essential oils. Jasminum is a genus of flowering plants, commonly used in the perfume industry and for ornamental purposes because of its bright flowers and unique fragrance. Mansour et al. (2022) prepared nanoemulsion formulations (NEs) of *Jasminum humile* and *Jasminum grandiflorum* essential oils and studied their cytotoxic and antiviral activities. The authors performed GC–MS analysis, and twenty-four compounds were detected in the EO of *J. humile* and seventeen compounds in the EO of *J. grandiflorum*. Biological investigations of pure EOs revealed weak cytotoxic and antiviral effects. Nevertheless, their NE demonstrated a higher biological value as cytotoxic and antiviral agents. Nanoemulsion formulations also showed realistic selectivity index for the viral-infected and cancer cells (particularly HepG-2) than normal Vero cells. According to the authors’ study, the outcomes demonstrated the significant dramatic effect of nanoemulsion preparation on the biological activity of EOs and other liposoluble phytopharmaceuticals. These in vitro findings suggest further in vivo and clinical investigations of the phytopharmaceutical nanoemulsion formulations as an effective approach to enhance the essential oils cytotoxic and antiviral activities.

Endophytic fungi represent a potentially useful and untapped reservoir of biologically active secondary metabolites with potential for application in the pharmaceutical industry [3,4]. In the study by Mohamed et al. (2022), a total of 25 fungal strains associated with stem parts of *Malus domestica* trees were isolated. Those isolates were identified based on the phenotypic and morphological combination and represented four genera. These isolated fungi were further fermented in a solid rice medium, followed by extraction with ethyl acetate (EtOAc). Next, the antimicrobial activity of those crude extracts was assessed against a variety of pathogenic bacteria. As a result, one endophytic fungal isolate demonstrated the highest activity and was chosen for further investigation by the authors. Based on its phenotypic, ITS ribosomal gene sequences, and phylogenetic characterization, this isolate was identified as *Aspergillus tubingensis* strain AN103. The authors then performed chemical investigation of its fermented culture, and as a result, four compounds were isolated and identified as Pyranonigrin A (1), Fonsecin (2), TMC 256 A1 (3), and Asperazine (4). The isolated compounds exhibited moderate-to-potent antibacterial activities against *Pseudomonas aeruginosa* and *Escherichia coli*, respectively. The antifungal activity results show that (3) and (4) had the maximum effect against *Fusarium solani* and *A. niger*, and (2) had the best effect against *Candida albicans*. Moreover, in a cytotoxicity assay against mouse lymphoma cell line L5178Y, (4) exhibited moderate cytotoxicity, whereas (1–3) showed weak cytotoxicity. These compounds might have potential as lead compounds to develop future cytotoxic and antimicrobial drugs (Contribution 2).

Malaria is a potentially life-threatening disease caused by the parasite *Plasmodium malariae*. Despite the current antimalarial drugs used to control infection, there is an emergence resistance which makes searching for effective, safe, and affordable alternatives an urgent necessity. In the work of Elmaidomy et al. (2022) on the widespread green seaweed *Halimeda macroloba*, two new compounds, along with four known compounds, were isolated and identified based on several NMR techniques and MS analysis. The authors performed extensive machine learning, and as a result, the cytochrome-C enzyme was found to be a potential antimalarial target for one of the isolated compounds. Docking, absolute binding free energy (ΔG binding), and molecular dynamics simulation (MDS) of this compound demonstrated a strong binding interaction with cytochrome-C. In vitro testing for the crude extract and isolated compounds revealed the potential inhibitory activity of both the extract and one of the isolated compounds against *P. falciparum*. The crude extract was able to inhibit the parasite growth with an IC_50_ value of 1.8 ± 0.35 µg/mL. Some of the isolated compounds showed varied degrees of inhibition, and their skeletons might be considered for the future development of new antimalarial agents (Contribution 3).

Invasive alien species represent one of the main contemporary environmental problems. In this regard, the roots of the invasive *Xanthium spinosum L.* were the focus of the study by Baldi et al. (2022). The authors concentrated on studying the ecological role of the invasive *Xanthium spinosum*, through the analysis of its volatile root metabolites and the evaluation of their allelopathic potential, with the ambition of preserving biodiversity and offering a long-lasting solution to the economic and ecological issues raised by invasive plant species. In their investigation, three sesquiterpene lactones with a 12,8-guaianolide skeleton, ziniolide, xantholide B (11α-dihydroziniolide), and 11β-dihydroziniolide, were identified from the essential oil, hydrosol extract, and hexane extracts from *Xanthium spinosum* L. The authors reported the ^1^H- and ^13^C-NMR data of 11β-dihydroziniolide, which was observed as a natural product for the first time in their study. Furthermore, the authors studied the allelopathic effects of *X. spinosum* root’s volatile metabolites on seed germination and seedling growth (leek and radish). Essential oils, as well as hydrosol- and microwave-assisted extracts, inhibited germination and seedling growth. The authors deduced that ziniolide derivatives appear to play a significant role in allelopathic interactions and could be the key to comprehending the invasive mechanisms of weeds; however, further investigations are required to ascertain the possible use of *Xanthium spinosum* as a natural herbicide (Contribution 4).

Antibiotic resistance among pathogenic bacterial isolates is considered a critical public health concern owing to the high number of patients suffering from incurable serious infections. One of the main contributors to hospital-acquired infections is the opportunistic, Gram-negative, widely distributed pathogenic bacteria *Pseudomonas aeruginosa*, which can result in a wide range of antibiotic-resistant infections that are accompanied by high rates of morbidity and mortality. Targeting this pathogenic bacteria, Abdel Bar et al. (2022) initiated their study on *Pelargonium × hortorum*, one of the most popular ornamental plants. The authors first traced the presence of catechin, gallic acid, scopoletin, and umckalin in different parts of two *Pelargonium × hortorum* cultivars. They then compared the obtained results with those of the root extract of the medicinal plant, *P. sidoides*, using the HPLC-UV technique. Traditionally, *Pelargonium sidoides* has been used to treat a wide range of disorders, including upper respiratory tract infections, such as bronchitis, the common cold, and sinusitis. The main constituents of *P. sidoides* showed antibacterial and antiviral activities against several respiratory pathogens. The comparative study revealed the presence of catechin and gallic acid in high concentrations in *Pelargonium × hortorum* cultivars. The investigation focused on the antibacterial activity of the methanolic extracts of different parts of *Pelargonium × hortorum* cultivars, as well as their major biological markers against 19 clinical isolates of *P. aeruginosa*. The anti-biofilm and anti-quorum-sensing (QS) potential of the tested biological markers were also studied by the authors. Moreover, the detailed mechanism of action was investigated using qRT-PCR and in silico docking studies against the main targets involved in the coordination of QS in *P. aeruginosa*. The outcomes of this investigation revealed the potential biofilm and quorum-sensing-inhibitory activity of the catechin and gallic acid combination as a novel alternative to inhibit bacterial pathogenicity (Contribution 5).

One prevalent skin disease that can affect people with all different types of skin is skin hyperpigmentation. Prof. Shimizu group assessed the melanin production inhibition ability of bamboo shoot skin (*Phyllostachys pubescens*), a noteworthy agricultural waste, with the goal of identifying natural products for skin hyperpigmentation. *P. pubescens* is a widely dispersed species in Japan. The authors in this study evaluated the total methanolic extract of bamboo peel extract for its skin-protective effects via measuring its melanin inhibitory activity and its suppression activity on the expression of tyrosinase mRNA levels. The authors measured melanin production in mouse melanoma cells and in a three-dimensional human skin model after the application of bamboo peel extract. As a result, the bamboo peel extract decreased melanin production in melanoma cells and in the three-dimensional human skin model. For further confirmation of the activity, the authors examined tyrosinase mRNA levels using real-time PCR analysis; notably, as reported in the study, the expression of tyrosinase mRNA level was sharply decreased. Their results demonstrated the effectiveness of bamboo peel extract in the inhibition of melanin synthesis. To find the bioactive compounds responsible for such activity and with the aim of isolating skin whitening agents, the authors performed a chemical study; several compounds have been isolated and identified by different techniques. The effect of the isolated compounds on the production of melanin, hyaluronic acid, and collagen was further studied. As reported by the authors, several compounds were found to decrease melanin production and increase collagen and hyaluronic acid production in the cells. Based on these interesting findings, bamboo shoots are of great value for consideration as a potential natural medicine for skin hyperpigmentation and related disorders (Contribution 6).

Microbial biotransformation is an effective experimental technique for modifying the structures of both natural and synthetic compounds [5]. It is used to either enhance the biological activity of the parent compound or to decrease the compound’s toxicity. It is an alternative approach to chemical reactions. One of the main advantages of microbial transformation is the selective addition of functional groups to the carbon skeleton, resulting in the production of new metabolites, which are challenging to obtain through chemical reactions. With the intention of improving the anti-inflammatory activity of vulgarin and investigating the metabolism of vulgarin by liver enzymes, ElGamal et al. (2023) performed the biotransformation of vulgarin. From the ground aerial parts of *Artemisia judaica* L. (Asteraceae), the authors isolated the eudesmanolide-type sesquiterpene lactone, vulgarin, and identified it using different techniques. Using the filamentous fungus, *Aspergillus niger* (ATCC 10549)*,* in the biotransformation process, three interesting metabolites were produced: 1-epi-tetrahydrovulgarin and unresolved C-1 epimeric mixtures of 1α,4α-dihydroxyeudesm-2-en-5αH,6,11βH-6,12-olide, in a ratio of 4:1. Two of the resulting metabolites were reported for the first time as new compounds. Vulgarin and its metabolites were evaluated as anti-inflammatory agents using the human cyclooxygenase (COX) inhibitory assay. The results showed that one of the metabolites retained selective inhibitory activity against COX-1. Additionally, the authors concluded the necessity of the presence of the α, β-unsaturated carbonyl group in vulgarin for better COX-2 inhibitory activity (Contribution 7).

*Hericium erinaceus* (Lion’s mane) is a medicinal mushroom that has been associated with several health benefits. It is well known that this mushroom stimulates neurotrophic factors, including nerve growth factor (NGF) and brain-derived neurotrophic factor (BDNF). Hericenone C, one of the major metabolites in the fruiting bodies of this mushroom, is known for its significant role in stimulating the production of nerve growth factors (NGFs) in brain astrocytes. Chemically, hericenone C is a meroterpenoid with a palmitic acid side chain. This fatty acid side chain seems highly susceptible to lipase decomposition under in vivo metabolic conditions; therefore, the neuroprotective activity of hericenone C seems to be attributed to the hydrolyzed product rather than the intact compound. To examine this hypothesis, the authors studied this phenomenon. In their study, the authors isolated hericenone C from the ethanol extract of the fruiting bodies; then, the isolated compound was treated with pancreatic lipase to cleave its fatty acid side chain; and finally, the plausible in vivo bioactive form of hericenone C was isolated. The authors analyzed the resulting compound and identified it using LC-QTOF-MS combined with ^1^H-NMR analysis. The resulting compound was named deacylhericenone. Then, the authors compared the biological activity of the neuroprotective properties of hericenone C and deacylhericenone. The lipase-derived compound was found to have higher BDNF mRNA transcription in human neuroblastoma cells (SH-SY5Y) and human colon cancer cells (Caco-2), and increased cell viability in the oxidative-stress-induced astrocytoma cell (1321N1) bioassay, compared with its parent compound. The outcomes of this study provide strong evidence that the neuroprotective activity of hericenone C must, in fact, be attributed to its derivative, deacylhericenone. Confirming the compound’s effectiveness for possible use as a nootropic agent requires more research on the mechanism underlying its neuroprotective activities and its bioavailability in vivo (Contribution 8).

This Special Issue also received two interesting review contributions. The first of these discussed the therapeutic benefits of mushroom polysaccharides, along with the advances made in the application of these compounds in cancer treatment. The review covered the clinical trials that have been documented using mushroom polysaccharides and the progress made through them. The difficulties facing the further extending of mushroom polysaccharides into cancer mycotherapy and the potential future applications of this class of compounds were also addressed in this review (Contribution 9).

The second review article covers the pharmacological properties and bioactive components in one of the leguminous plants: *Retama monosperma* L. (Boiss.) or *Genista monosperma* L. (Lam.). *R. monosperma* has been widely used in folk medicine and has attracted considerable interest due to its wide range of pharmacological properties and antioxidant, anti-aging, antibacterial, antifungal, anti-inflammatory, antiproliferative, antitumoral, as well as antileukemic activities. The authors critically emphasized the beneficial effects of flowers, stems, seed extracts, and isolated compounds from *R. monosperma* in health care, industrial, and other applications. The authors also discussed how these compounds might be used as potential candidates for future drug discovery (Contribution 10).

In the light of the published papers that comprise this Special Issue, we believe that our objectives have been achieved. We appreciate all the contributions that were received, and hope that all *Molecules* readers will find the papers interesting. Finally, there is still much more that natural products can do for humanity. We wish all authors and readers the best of luck and insightful discoveries in their future studies.
